# Resolving Cypriniformes relationships using an anchored enrichment approach

**DOI:** 10.1186/s12862-016-0819-5

**Published:** 2016-11-09

**Authors:** Carla C. Stout, Milton Tan, Alan R. Lemmon, Emily Moriarty Lemmon, Jonathan W. Armbruster

**Affiliations:** 1 Department of Biological Sciences, Auburn University, 101 Rouse Life Sciences Building, Auburn, AL 36849 USA; 2 Department of Scientific Computing, Florida State University, Tallahassee, FL 32306 USA; 3 Department of Biological Sciences, Florida State University, Tallahassee, FL 32306 USA

**Keywords:** Fish, High-throughput sequencing, Phylogenetics, Ostariophysi, Cyprinidae

## Abstract

**Background:**

Cypriniformes (minnows, carps, loaches, and suckers) is the largest group of freshwater fishes in the world (~4300 described species). Despite much attention, previous attempts to elucidate relationships using molecular and morphological characters have been incongruent. In this study we present the first phylogenomic analysis using anchored hybrid enrichment for 172 taxa to represent the order (plus three out-group taxa), which is the largest dataset for the order to date (219 loci, 315,288 bp, average locus length of 1011 bp).

**Results:**

Concatenation analysis establishes a robust tree with 97 % of nodes at 100 % bootstrap support. Species tree analysis was highly congruent with the concatenation analysis with only two major differences: monophyly of Cobitoidei and placement of Danionidae.

**Conclusions:**

Most major clades obtained in prior molecular studies were validated as monophyletic, and we provide robust resolution for the relationships among these clades for the first time. These relationships can be used as a framework for addressing a variety of evolutionary questions (e.g. phylogeography, polyploidization, diversification, trait evolution, comparative genomics) for which Cypriniformes is ideally suited.

**Electronic supplementary material:**

The online version of this article (doi:10.1186/s12862-016-0819-5) contains supplementary material, which is available to authorized users.

## Background

Cypriniformes (minnows, carps, loaches, and suckers) is the largest group of freshwater fishes in the world. Diversity ranges from some of the smallest vertebrates in the world (*Paedocypris*, 7.9 mm in standard length) to members of *Tor* (almost 3 m SL) [1]. The number of valid species is currently estimated at around 4300 [2] with as many as 2500 still awaiting description [3]. To place the Cypriniformes into perspective, about one third of freshwater fish species is a cypriniform and about 6 % of all vertebrate species is a cyprinform [2]. Species of Cypriniformes are distributed in freshwater habitats across Asia, Europe, Africa, and North America [4]. Example representatives include the zebrafish (*Danio rerio*), a model organism used in genomic and developmental biology, important aquaculture species like the common carp (*Cyprinus carpio*), major invasive species to North America such as *Hypophthalmichthys* (silver carp), and many popular aquarium species (rasboras and barbs).

For taxonomic clarity, this study follows the proposition by Mayden and Chen [1] that elevates subfamilies within Cyprinidae to the family level based on consistent support of major clades. Superfamilies are elevated to the suborder level to be consistent with the recognition of suborders as the taxonomic level above family and below order in the classification of bony fishes [5, 6]. Other taxonomic assignments follow designations established by Tang et al. [7], Kottelat [8], van der Laan et al. [9] and Yang et al. [10]. Because of the great diversity within Cypriniformes, most phylogenetic studies have focused on smaller groups within the order (for example [11–14]). Approaches used to resolve relationships at these levels have typically included standard methods using PCR to amplify targeted mitochondrial and/or nuclear genes [11–19]. These approaches have had varied success at elucidating relationships at these taxonomic levels, but deeper, all-inclusive studies have resulted in conflicting phylogenies. These major differences in findings even include two publications in the same volume [1, 19] whose results are incongruent. Morphological studies have also been at odds with the molecular hypotheses, particularly concerning placement of the paedomorphic taxa (*Danionella, Paedocypris,* and *Sundadanio*) [1, 20–22]. The results of analyses to date mean that this radiation of organisms that is nearly the size of the Mammalia and that is the predominant freshwater order of fishes has an unsettled taxonomy and phylogeny despite the fact that it has been very highly studied. With the vertebrate developmental model (zebrafish) being part of the Cypriniformes, we are currently lacking a basic understanding of the evolutionary context of its characteristics, and it is clear that new approaches to the phylogenetics of this very important group of fishes must be employed.

To date, the only nuclear genomic scale study [23] consisted of 100 genes and was limited to only thirteen individuals, most of which belong to Xenocyprididae within Cyprinoidei. The large number of taxa in Cypriniformes has forced researches to either focus on a small subset of representatives with an increasing number of molecular loci, or focus on large taxonomic representation with relatively fewer numbers of markers.

Evaluating tree topologies from previous large-scale studies has led to moderate consensus supporting monophyly for some clades within the order, including families of loaches (e.g. Botiidae, Cobitidae, Balitoridae, Nemacheilidae), Catostomidae (suckers), Cyprinidae, Xenocyprididae, Gobionidae, Leuciscidae, and Acheilognathidae [1, 19, 24–33]. Despite support for monophyly of many families, clear establishment of the relationships among them still remains elusive. Other families, most notably Danionidae, have been more problematic, with paedomorphic genera like *Paedocypris* and *Sundadanio* changing placement across trees employing both morphological and varying molecular data [1, 19–21, 31, 34].

If analyses result in incongruent relationships due to conflict or weak phylogenetic signal among individual genes, the next approach to establishing robust resolution would be to incorporate high-throughput sequencing data that can increase the signal to noise ratio and reduce stochastic error. New methods have been established that have been specifically tailored for use in systematics [35–37] and that address problems typical of transcriptome approaches for phylogenomics. These problems include tissue preservation, orthology assessment, missing data, and resolution capabilities across various taxonomic levels [35–37]. All of these factors make anchored hybrid enrichment an attractive option for addressing the phylogenetic uncertainties still present within Cypriniformes. This study represents the largest dataset developed for Cypriniformes, both in taxonomic representation and genetic data, ameliorating many of the problems associated with resolving the relationships among and within families of this order. Not until these relationships are resolved can researchers begin to take advantage of the size, diversity, and distribution of Cypriniformes to gain insight into various biological facets, such as biogeography, timing of diversifications, morphological and ecological evolution, and comparative genomics.

## Methods

### Taxon selection and tissue preparation

The 172 taxa selected for this study (Additional file 1: Table S1) represent almost all families within the order. Families not represented in this study are: Psilorhynchidae (26 species), Barbuccidae (two species), Tincidae (13 species), Serpenticobitidae (three species), Ellopostomidae (two species) and Leptobarbidae (five species). Species were chosen based on tissue availability and because of their incorporation in recent studies that will allow for direct comparisons [11, 13, 14, 26, 30, 38, 39]. Type genera for each of the families were included if available. Exceptions include Botiidae, Balitoridae, Gastromyzontidae, and Xenocyprididae, but in these cases other representatives were chosen based on their supported inclusion within their respected families according to previous studies [8, 40]. Three outgroup taxa were chosen to represent the three other ostariophysan orders: Siluriformes, Gymnotiformes, and Characiformes.

Whole genomic DNA was prepared using the Omegabiotek E.Z.N.A. animal tissue extraction kit (product #D3396-02) and verified for quality and quantity using gel electrophoresis and nanodrop, respectively.

### Locus selection and probe design

Although the Anchored Hybrid Enrichment kit developed for vertebrates by Lemmon et al. [36] contains a fish reference (*Danio*) and has been utilized in teleosts with moderate success [41], we desired an enrichment tool more efficient and appropriate for phylogenomics in teleosts. Because of the complex nature of teleost genome evolution, which involved multiple whole-genome duplications and lineage-specific gene losses [42], it is impractical to identify a set of loci that are truly single-copy across all of Teleostei. Previous studies claiming to have identified single-copy loci in teleosts (e.g. [43]) likely only identified loci that were single-copy in the species they considered; evaluation of those loci in additional teleost lineages suggests that these loci are not universally single-copy (see below). Consequently, we aimed to target loci containing up to four gene copies in each of three diverse lineages of teleosts: zebrafish, platyfish, and cichlids.

Candidate target regions for Teleostei were derived by combining the 394 Vertebrate Anchor (v2) loci of Prum et al. [44] and the 135 loci identified as Fugu-*Danio* single-copy orthologs by Li [43]. For the vertebrate anchor loci, teleost orthologs were obtained for *Danio rerio* (danRer7) using the human (hg19) coordinates and the USCS genome browser batch-coordinate (liftover) tool [45]. For the Fugu-*Danio* orthologs, orthologous human (hg19) and chicken (galGal3) coordinates were obtained using the USCS liftover tool and the *Danio* coordinates identified by Li [43]. Once the coordinates for *Danio*, *Homo*, and *Gallus* were obtained for all 529 candidate target regions, sequences corresponding to those regions [plus sufficient flanking region to obtain up to 3000 base pairs (bp) total] were extracted from the genomes and aligned by locus using MAFFT [46], v7.023b with “–genafpair” and “–maxiterate 1000” flags. The alignments were then used to generate a *Danio*-specific reference database containing spaced 20-mers. The *Danio* reference was then used to identify homologous regions in the genomes of zebrafish (Cypriniformes: Cyprinidae: *Danio rerio*; danRer7), platyfish (Cyprinodontiformes: Poeciliidae: *Xiphophorus maculatus* [47], and cichlid (Perciformes, Cichlidae: *Maylandia zebra*; [48]).

As expected, we obtained multiple homologs for many of the candidate loci (only 64 loci were single copy in all three species). Consequently, only 277 loci had fewer than five homologs per species and were considered further. We aligned with MAFFT [46], v7.023b with “–genafpair” and “–maxiterate 1000” flags) all homolog sequences (up to 12 per locus) for each of the 277 candidates together with the homologous human probe region sequence from the Vertebrate Anchor (v2) design. Alignments were then manually inspected for misplaced and grossly misaligned sequences, which were removed. Finally, alignments were trimmed to include regions best suited for Anchored Hybrid Enrichment (conserved, low-gap, high taxon representation), taking care that the chosen region contained the human probe region. A total of 260 loci were retained.

Finally, in order to ensure efficient enrichment, we checked for high-copy regions (e.g. microsatellites and transposable elements) in each of the three teleost references as follows. First, a database was constructed for each species using all 15-mers found in the trimmed alignments for that species. We also added to the database all 15-mers that were 1 bp removed from the observed 15-mers. The genome for the species was then exhaustively scanned for the presence of these 15-mers and matches were tallied at the alignment positions at which the 15-mer was found. Alignment regions containing > 100,000 counts in any of the three species were masked to prevent probe tiling across these regions. Probes of 120 bp were tiled uniformly at 5.5× tiling density.

### Data collection

Multilocus sequence data were collected at the Center for Anchored Phylogenomics at Florida State University (www.anchoredphylogeny.com) following Lemmon et al. [41] with some adjustments. Each genomic DNA sample was sonicated to a fragment size of ~175–300 bp using a Covaris E220 Focused-ultrasonicator with Covaris microTUBES. Library preparation and indexing followed Meyer and Kircher [49]. Indexed libraries were pooled at equal quantities (12 pools of 16 samples each), and the library pools were enriched using a custom Agilent Custom SureSelect kit (Agilent Technologies), with probes designed as described above. The 12 enriched library pools were pooled with equal quantities for sequencing on 4 PE150 Illumina HiSeq2000 lanes with 8 bp indexing. Sequencing was performed at Florida State University in the College of Medicine Translational Science Laboratory.

### Data analysis

Reads were quality filtered using Illumina’s Casava software with the chastity filter set to high. In order to increase read length and accuracy overlapping reads were then merged following Rokyta et al. [50]. Non-overlapping read pairs were kept separate but still used in the assembly. All reads were then assembled into contigs following Prum et al. [44] using mapping references derived from the zebrafish, platyfish, and cichlid sequences used for probe design. This assembler produces separate contigs for gene copies differing by more than 5 % sequence divergence. To reduce errors caused by low-level indexing errors during sequencing, contigs were then filtered by removing those derived from fewer than 50 reads. Additional file 2: Table S2 provides a summary of the sequence data collected and assemblies that resulted.

Sets of homologs were produced by grouping by target locus (across individuals) and the filtered consensus sequences. Orthology was then determined for each target locus as follows. First, a pairwise distance measure was computed for pairs of homologs, with distance being computed as the percentage of 20-mers observed in the two sequences that were found in both sequences. A neighbor-joining clustering algorithm was then used to cluster the consensus sequences in to orthologous sets, with at most one sequence per species in each orthologous set (see [44] for details). In order to minimize the effects of missing data, clusters containing fewer than 130 (72 %) of the species were removed from downstream processing.

Sequences in each orthologous set were aligned using MAFFT v7.023b [46] with --genafpair and --maxiterate 1000 flags. In order to remove poorly aligned regions raw alignments were then trimmed and masked following Prum et al. [44], with the following adjustments: sites with > 50 % similarity were identified as good, 20 bp regions containing < 14 good sites were masked, and sites with fewer than 30 unmasked bases were removed from the alignment.

For all phylogenetic analyses, sequences from the gymnotiform, siluriform, and characiform species were used as the outgroup. For the concatenated dataset, the alignment was partitioned by locus and the phylogeny estimated using RAxML using GTR+ Γ model with 500 bootstrap replicates. For the species tree analysis, a maximum likelihood phylogeny was estimated with 100 bootstrap replicates for each of the separate loci using RAxML with GTR+ Γ model assumed. We then used the RAxML bootstrap trees to estimate a species tree using STAR [51] with default parameters using STRAW [52]. ASTRAL-II (v4.10.2) [53] was also used for species tree inference using the gene trees and their 100 bootstrap replicates. We performed 100 replicates of multi-locus bootstrapping.

To test our analyses against previous morphological hypotheses, we re-examined the datasets in Conway [54] and Britz et al. [21] by running 1000 replicates of a heuristic search in PAUP* [55]. We traced the characters in Mesquite v.3.04 [56]. We also performed Bayesian analyses on these morphological datasets under the Mk + Γ model in mrBayes 3.2 [57], which has been demonstrated to perform better than parsimony due to rate heterogeneity in character evolution [58]. Estimating rate heterogeneity can be biased by sampling only variable or parsimony-informative characters, so we analyzed the data with correction for parsimony-informative characters for the Conway [54] dataset and variable characters for the Britz et al. [21] datasets (one character in these datasets was not parsimony-informative). For each dataset, we ran MCMC with two runs of four chains for 1,000,000 generations, sampling every 1,000. We assessed convergence using Tracer v1.5 [59].

## Results

**Fig. 1:**
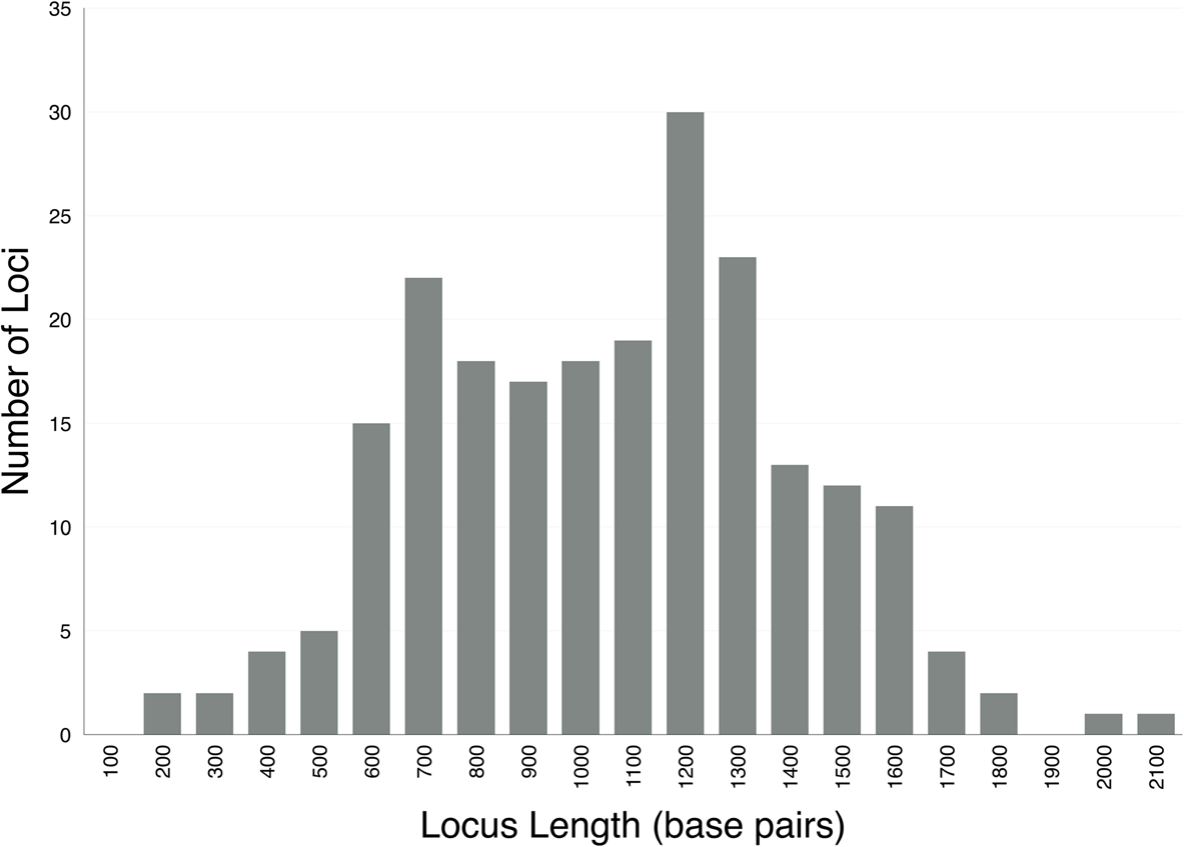
Histogram showing lengths of loci in base pairs

**Fig. 2:**
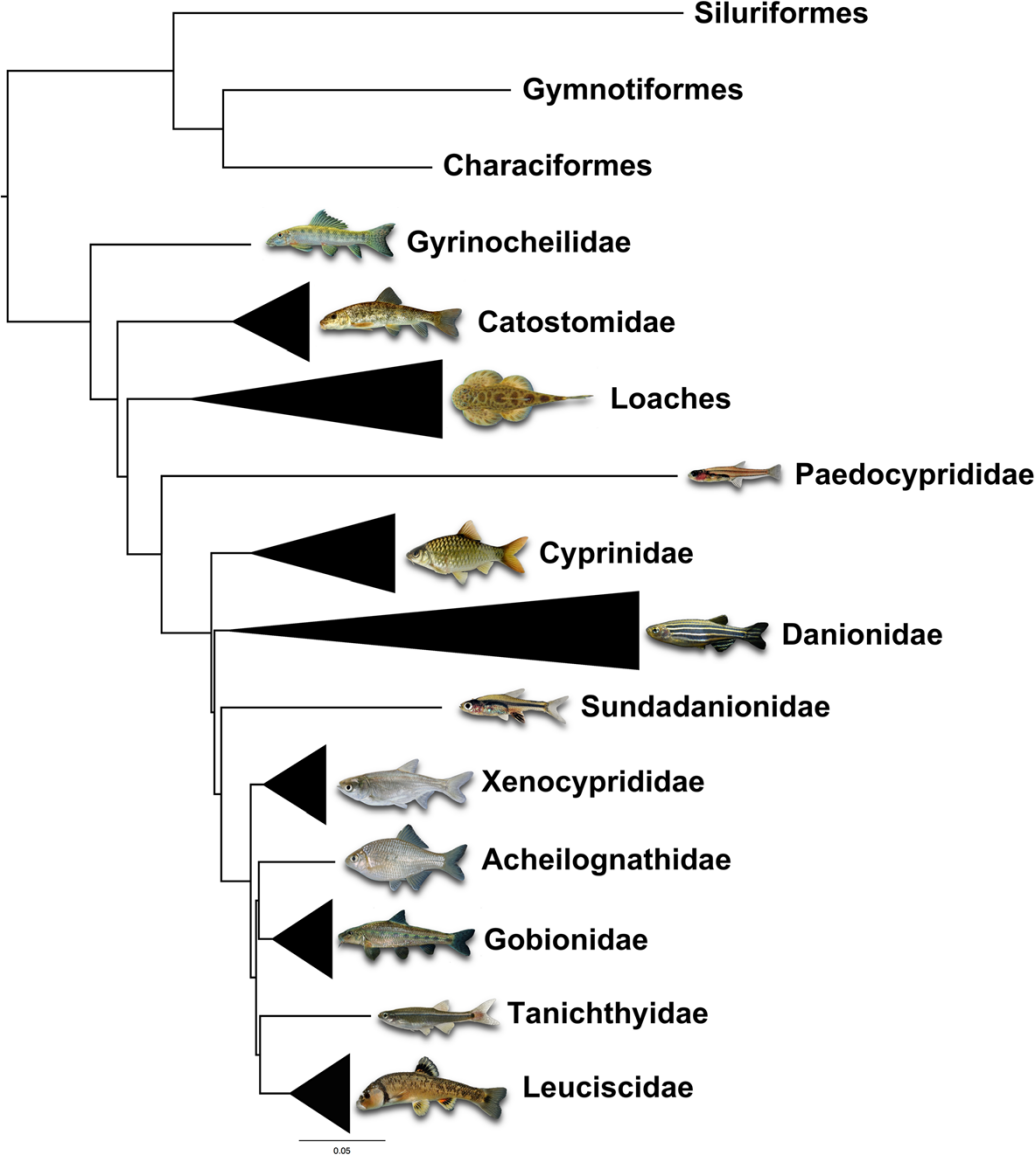
Maximum likelihood tree based on concatenation of all specimens collapsed into major clades. For this and all subsequent tree figures, all nodes shown are 100 % bootstrap supported unless otherwise indicated, and the scale bar represents the number of nucleotide substitutions per site

A total of 315,288 base pairs (bp) spanning 219 loci were obtained for use in estimating the phylogenetic relationships. Average locus length was 1011 bp with a range of 134–2119 bp (Fig. 1). The total number of informative characters was 295,252 bp with only 3.48 % missing data (Dryad accession link: doi:10.5061/dryad.b3d03; raw reads available on NCBI SRA (Bioproject PRJNA345212). Our results show promise for the ability of this method to provide robust support for relationships, with 97 % of nodes resolved at 100 % bootstrap support (Additional file 3: Figure S1). Findings include resolution of major clades supported by previous work (e.g. families within Cyprinoidei — see Fig. 2), but relationships among these clades differ. Major results include paraphyly of Cobitoidei, with Gyrinocheilidae sister to the rest of Cypriniformes, followed by Catostomidae sister to the remaining ingroup (see below). We find support for Mayden and Chen’s [1] recognition of Paedocyprididae and Sundadanionidae since neither is recovered within Danionidae. Leuciscidae are sister to Tanichthyidae, Acheilognathidae are sister to Gobionidae, and these two clades are sister to each other [(Acheilognathidae + Gobionidae) + (Tanichthyidae + Leuciscidae)]. Xenocyprididae falls sister to these four families.

### Concatenated tree vs. species tree

We find only a few major differences between our maximum likelihood concatenated tree (CT) and the species trees (ST; Additional file 4: Figure S2 and Additional file 5: Figure S3). These include support for monophyly of Cobitoidei in the ST but not in the CT, and a different placement for the Danionidae between the two trees. Other minor differences are found among a few shallow sister relationships that had lower support values in both trees. Other studies have shown that concatenation methods may perform better over coalescent species tree methods, especially at deeper nodes, and our discussion of clades will focus on the CT tree [44, 60, 61].

### Reanalysis of Cobitoidei morphological datasets

The most robust morphological phylogenies putatively supporting a monophyletic Cobitoidei is that of Conway [54]; however, when we reanalyzed the characters using parsimony in PAUP* [55], we achieved different results. We ran the analysis according to Conway [54] with the exception that we ran 1000 replicates of a heuristic search; it appears Conway [54] only ran a single replicate of a heuristic search, and that search settled on a tree island of 14 most parsimonious trees. We found one additional tree island with an additional 56 trees, which was found nearly as often as the 14-tree island (515 times vs. 485). The strict consensus of the 70 trees showed a polytomy at the base of the Cypriniformes with the gyrinocheilids, catostomids, loaches, and cyprinoids. The analyses in Britz et al. [21] did use 10 replicates of the heuristic search and are more accurate (we found more trees for their Morphological Dataset 3), and always found a monophyletic Cobitoidei, but this was weakly supported. Conway [54] lists seven characters supporting Cobitoidei, but our analysis showed that two of these (characters 32:1 and 99:1) were not listed as changed along the branch leading to the Cobitoidea and only one (character 19:1) is actually present in all families of cobitoids. All the remaining derived character states are absent in one of the three lineages (gyrinocheilids, catostomids, or loaches) meaning morphological support for a monophyletic group containing these three clades is poor. Support was stronger for a sister group relationship between gyrinocheilids plus catostomids (seven characters in [54], six in our analysis); however, we found seven characters supporting loaches plus cyprinoids (characters 7:0, 18:0, 46:1, 76:0, 83:2, 100:0, and 111:2) and seven characters supporting catostomids plus loaches plus cyprinoids (characters 11:0, 31:1, 36:1, 53:1, 68:1, 69:1, and 77:1) indicating roughly equal morphological support for the two hypotheses. Considerable homoplasy is found in most of the characters under all arrangements; however, characters 53, 83, and 77 provide unambiguous support for the relationships presented in this study.

In addition, the Bayesian analysis of the morphological characters resulted in only poor support (<.95 posterior probability, following Alfaro & Holder [62]) for monophyly of the Cobitoidei. In the analysis of the Conway [54] dataset, the catostmoids, gyrinocheilids, loaches, and cyprinoids form an unresolved polytomy in the consensus tree; this differs from the support present in Conway [54] for this node (.5–.9 pp). In the analyses of the Britz et al. [21] datasets, support ranged from .57 to.63 posterior probability across datasets, indicating low levels of support.

## Discussion

We have presented the first order-wide, phylogenomic analysis of the Cypriniformes, and we demonstrate the utility of anchored enrichment at assessing the relationships of fishes from deep to more recent divergences. Our analyses demonstrate conflict in the relationships of the Cobitoidei, the placement of *Paedocypris* as sister to all other cyprinoids, and a validation of the previously well-supported monophyly of many major cypriniform families. Although the wide variety of different hypotheses for the cypriniforms has been called the “Cypriniformes tree of confusion” [22, 63], the anchored enrichment phylogenomic tree that we present provides the most robust phylogenetic analysis to date, supporting many of the previous hypotheses of relationships and providing new ideas that will require further scrutiny.

### Non-monophyly of Cobitoidei

**Fig. 3:**
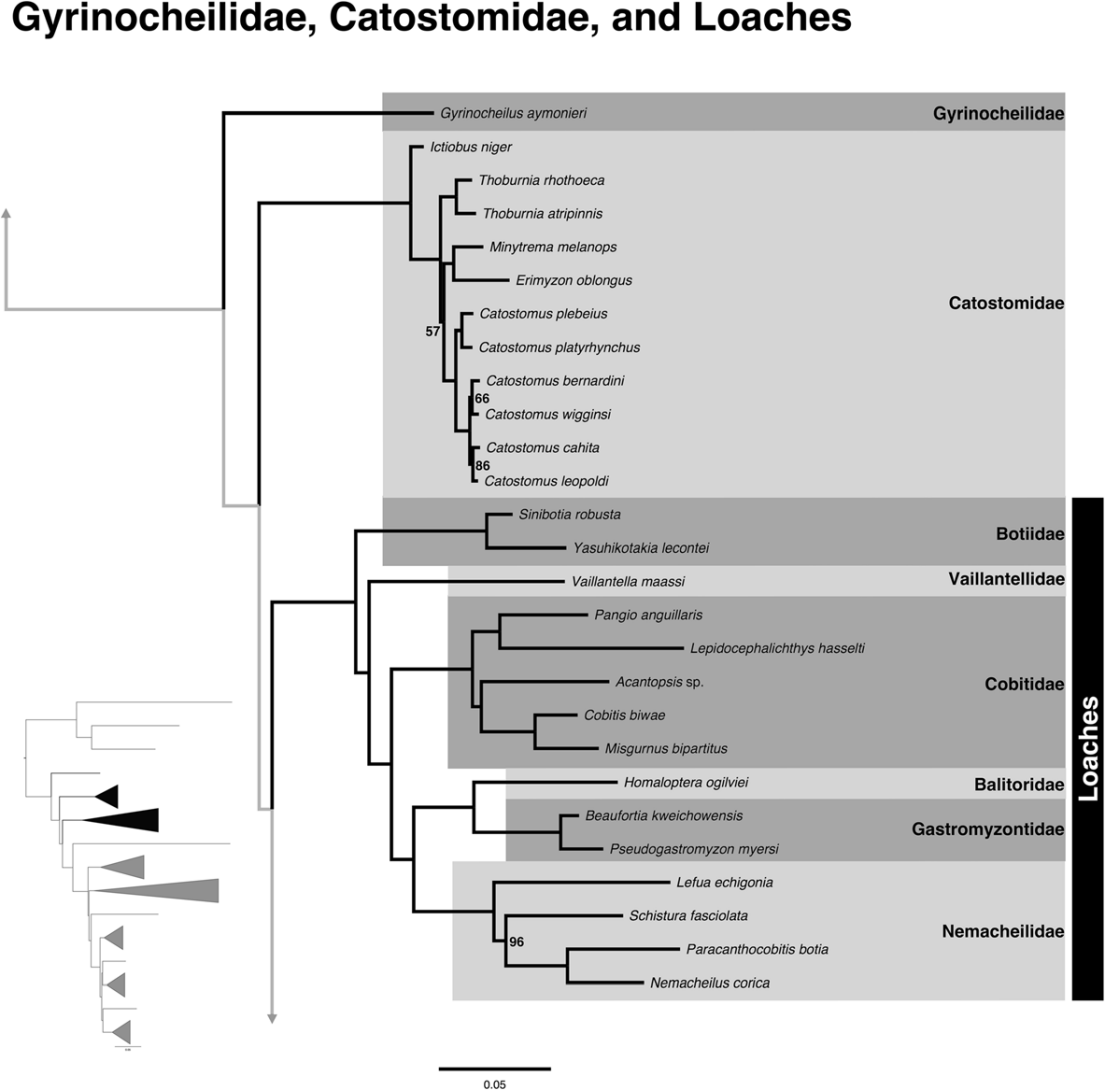
Expansion of Cobitoidei families from the ML tree shown in Fig. 1. All nodes are 100 % bootstrap supported unless otherwise indicated, and the scale bar represents the number of nucleotide substitutions per site

The most surprising result of the study is the non-monophyly of Cobitoidei in the concatenation analysis (Fig. 3). Cobitoids are largely believed to be monophyletic, however, many different placements of the taxa have been found. The Gyrinocheilidae (three species), Catostomidae (83 species), and loaches (Botiidae, 56 species; Balitoridae, 229 species; Cobitidae, ~198 species; Nemacheilidae, 658 species; Vaillantellidae, three species; and Gastromyzontidae, 137 species) represent successive sister groups to the Cyprinoidei in our concatenated analyses. Species tree analysis did find a monophyletic Cobitoidei; however, recent research has found that species tree analyses may not be as accurate at deeper levels of the phylogeny [44, 60, 61]. Considering these studies, the depth of the nodes leading to members of Cobitoidei, and the results of the reanalysis of morphological data that had previously supported monophyly of the group, we are compelled to follow the relationships presented in the concatenation analysis until further exploration regarding the discrepancies between concatenation versus species trees is conducted and consensus by the scientific community is reached.

Phylogenetic reanalysis of available morphological characters does not provide strong evidence for a monophyletic Cobitoidei, and morphological characters provide at least equally strong support for the relationships presented here. We restrict Cobitoidei to the loaches, and erect new suborders for the Gyrinocheilidae (Gyrinocheiloidei) and the Catostomidae (Catostomoidei).

### Cyprinidae

**Fig. 4:**
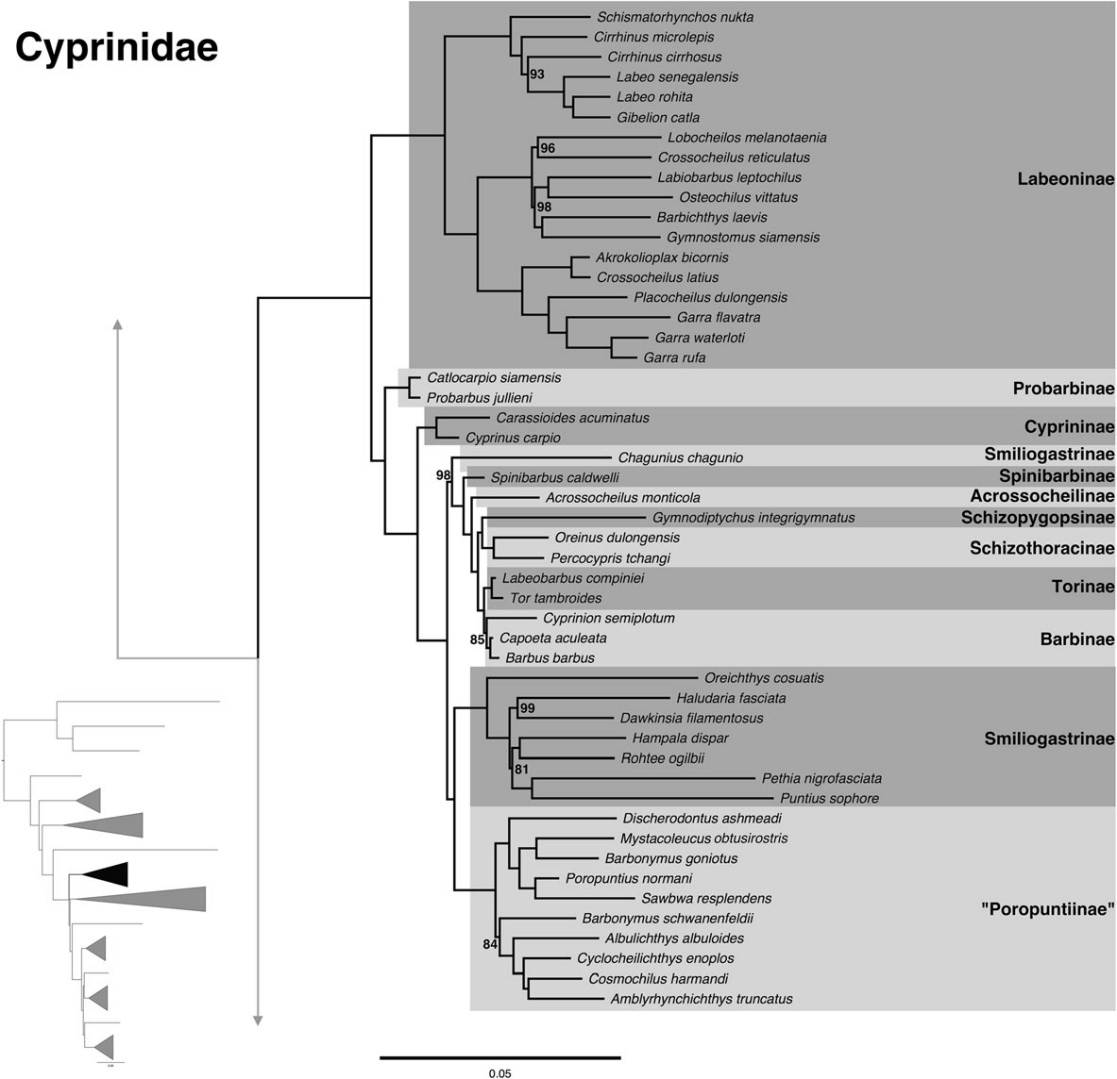
Expansion of Cyprinidae from the ML tree shown in Fig. 1 (inset) with subfamilies highlighted. All nodes are 100 % bootstrap supported unless otherwise indicated, and the scale bar represents the number of nucleotide substitutions per site

Among the Labeoninae (Fig. 4), we find support for many of the tribes (discussed as subtribes in Yang et al. [64]). These tribes, based on analysis of four nuclear and five mitochondrial genes, are: Labeonini, Garrini, “Osteochilini”, and “Semilabeonini” (quotation marks denote a lack of formal description). Labeonini was resolved as monophyletic as in Yang et al. [64]. We also obtained *Gibelion* nested within *Labeo*, and non-monophyly of *Cirrhinus*. Although Kottelat [8] recognized *Gymnostomus* as the valid generic name for *Henicorhynchus siamensis*, we find a pattern similar to Yang et al. [64] where this species is within the “Osteochilini” species group instead of with other members of *Gymnostomus* in Labeonini. *Placocheilus cryptonemus* was resolved as belonging to “Semilabeonini” in Yang et al. [64] but *Placocheilus dulongensis* in the AE tree is resolved within Garrini. Lothongkham et al. [65] established *Placocheilus* as a synonym of *Garra*, but members of this group need further study to determine which species should be synonymized with *Garra* (e.g. *P. dulongensis*). Because of the particular placement of *Placocheilus dulongensis* within Garrini (compared to other members of *Placocheilus* in “Semilabeonini”), our analyses did not include a representative of the “Semilabeonini” species group, but the relationships among the tribes of Labeoninae presented in this study are consistent with Yang et al. [64].

For the remaining members of Cyprinidae, we find resolution for clades similar to those by Yang et al. [10] although none of the AE relationships among these clades are consistent with their results. For example, we resolve Labeoninae as sister to remaining members of Cyprinidae as opposed to Probarbinae as presented in Yang et al. [10]. Of particular interest is *Chagunius chagunio,* which Yang et al. [10] placed in the Smiliogastrinae. We obtain it as sister to a clade comprised of Spinibarbinae, Acrossocheilinae, Schizopygopsinae, Schizothoracinae, Torinae and Barbinae, with other Smiliogastrinae species more closely related to “Poropuntiinae” than to *Chagunius*. Yang et al. [10] had 0.80 posterior probability support for their placement based on mitogenome data, but less than 0.50 in their nuclear analysis (RAG1). Yang et al. [10] found numerous inter-clade hybridization events leading to allopolyploidy, which greatly complicates phylogenetic analysis within the Cyprinidae. We leave *Chagunius* as *incertae sedis* within Cyprinidae.

### Danionidae, Paedocyprididae, Sundadanionidae

**Fig. 5:**
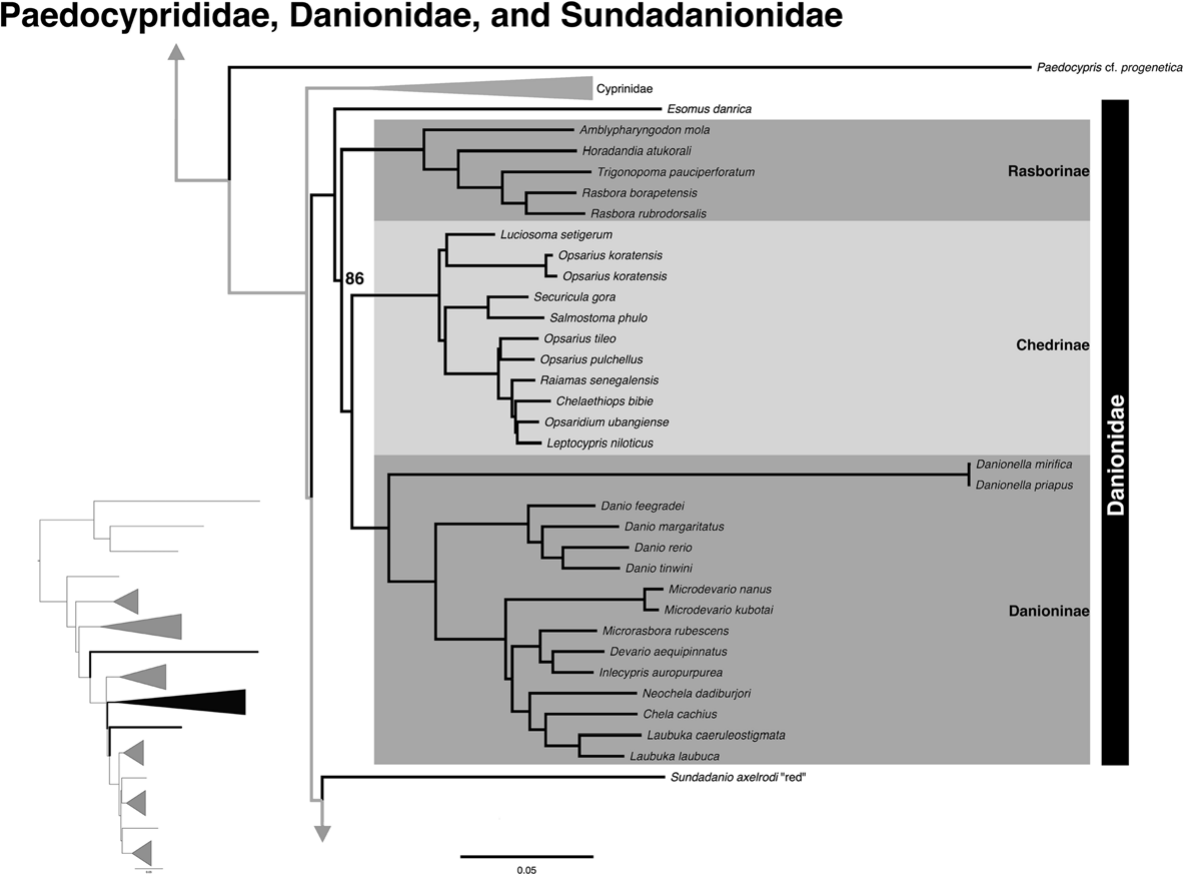
Expansion of Danionidae from the ML tree shown in Fig. 1 (inset) with subfamilies labeled. Also included are *Paedocypris* and *Sundadanio*, highlighting their placement outside of Danionidae. All nodes are 100 % bootstrap supported unless otherwise indicated, and the scale bar represents the number of nucleotide substitutions per site

We obtain three major groups that have previously been resolved in the Danionidae based on both morphological and molecular evidence: Danioninae, Chedrinae, and Rasborinae [11, 19, 31, 34, 66, 67] Fig. 5. Although support for monophyly of Danionidae has been reported with relatively low support in most prior studies (usually <70 % bootstrap support), we resolve Danionidae (minus *Paedocypris* and *Sundadanio*) as monophyletic with 100 % bootstrap support. Previous studies also provided poor or no support for the relationships between Danioninae, Chedrinae, and Rasborinae. We find robust support (100 % bootstrap support) for Rasborinae as sister to Danioninae plus Chedrinae.

Differing from previous studies, we find the genus *Esomus* as a separate lineage sister to all remaining members of the Danionidae. The placement of *Esomus* has been contentious [67]. *Esomus* has been placed as closely related to *Danionella* or *Sundadanio* within Danioninae with poor support [19, 31, 66]. Because of poorly supported nodes, molecular phylogenies are ambiguous on the placement of *Esomus* among the clades of Danionidae. Liao et al. [67] remark that *Esomus* has a long branch in molecular phylogenetic analyses, and this may attract this branch towards other long branches such as *Danionella* and *Sundadanio*. Using morphological characters, Liao et al. [67] establish *Esomus* as sister to all other members of Chedrinae based on four characters, including two acquired states and two homoplasious states. In a subsequent paper, they admit this topology is never recovered in molecular analysis [68]. Both of the acquired character states relate to the postcleithrum: first its presence, and secondly its orientation. In *Esomus*, the postcleithrum is absent, and the postcleithrum orientation was coded as missing, and thus may not be informative on its placement relative to the Chedrinae. Additionally, although postcleithrum absence within Danionidae is only found in the Chedrinae, postcleithrum absence is also found in disparate genera from multiple cyprinoid groups including leuciscids, cyprinids, and gobionids [67]. Morphological homoplasy, long branch attraction, and short intervening branch lengths between danionid clades may have all contributed to the varying placement of *Esomus* between molecular and morphological studies.

We do not find support for *Paedocypris* and *Sundadanio* within Danionidae. This conflicts with multiple phylogenetic studies [11, 31, 34, 66]. Prior molecular analyses placing *Paedocypris* and *Sundadanio* within Danionidae have poor to moderate support. Rüber et al. [34] obtain *Paedocypris* and *Sundadanio* as sister to Danionidae with 0.86 posterior probability (pp), less than the 0.95 pp required to be considered strong support [62]. Fang et al. [31] place *Paedocypris* and *Sundadanio* sister to other Danionidae, but with only 0.76 pp. Tang et al. [1] find *Paedocypris* and *Sundadanio* in a polytomy with Rasborinae and Danioninae in a moderately supported clade (76 % bootstrap in CT). Mayden and Chen [1] proposed the exclusion of these two genera from Danionidae, which resolve *Paedocypris* as either sister to Cypriniformes or Cyprinoidei, and *Sundadanio* as sister to a clade comprised of Xenocyprididae, Acheilognathidae, Tincidae, Tanichthyidae, Gobionidae, and Leuciscidae. Our results are congruent with their placement of *Sundadanio*, but we resolve *Paedocypris* as a lineage sister to the remainder of Cyprinoidei. This is incongruent with the published topology of Mayden and Chen [1], but is congruent with an equally supported topology and an unpublished mitogenome analysis as discussed in their study. Difficulty in obtaining consistent placement of *Paedocypris* and *Sundadanio* may be due to several factors. Britz et al. [21] reanalyzed the Rüber et al. [34] and Mayden and Chen [1] datasets and demonstrated that the phylogenetic signal in most previously sequenced genes are equivocal on the placement of *Paedocypris*. Additionally, the branches for *Paedocypris* and *Sundadanio* are quite long, potentially contributing to spurious results with limited datasets prior to high-throughput sequencing. Our results robustly support the recognition of Paedocyprididae and Sundadanionidae based on their independent lineages from the remaining members of Danionidae.

Britz et al. [21] provide considerable morphological support for the paedomorphs forming a monophyletic clade, even when using the dataset of Conway [54] that did not include characters specific to paedomorphs. We reanalyzed the dataset of Britz et al. [21], and found that even with their morphological dataset 3 (that of Conway [54], plus some additional taxa and only one species of *Psilorhynchus*), we noted three character changes supporting all paedomorphs as monophyletic and nine character changes uniting *Paedocypris* and *Danionella*. Adding in characters specific to the paedomorphs [morphological datasets 4 and 5 from Britz et al. [21] only increases the level of support. Under Bayesian analysis, the support for paedomorphic taxa forming a clade is weak in morphological dataset 3 (0.76 pp) but increases dramatically with addition of the paedomorphic-specific characters of datasets 4 and 5 (1.00 pp). We believe the weak support for the relationships of the various cyprinoids in the original dataset [54] explains the disparity between the morphological and molecular hypotheses. In both the parsimony and Bayesian reanalyses of Britz et al.’s [21] morphological dataset 3, the basal relationships of the cyprinoids are an almost complete comb. Without strong support for relationships within the Cyprinoidei, the dataset is insufficient for distinguishing synapomorphy from convergence among the paedomorphs, and adding characters specific to paedomorphs will only decrease the ability of the morphology to detect convergence. Conway’s [54] dataset already contains considerable homoplasy before the addition of the paedomorphs, indicating that morphological evolution within Cypriniformes was rapid. The support in our dataset for three separate transitions to paedomorphism is strong, corroborating Mayden and Chen’s [1] suggestion of convergence in morphology, and we find at least five character changes in the Britz et al.’s [21] morphological dataset 3 that support monophyly of the cyprinoids minus *Paedocypris* (21:1, 24:1, 34:1, 82:1, 101:0).

### Xenocyprididae, Acheilognathidae, Gobionidae, Tanichthyidae, and Leuciscidae

**Fig. 6:**
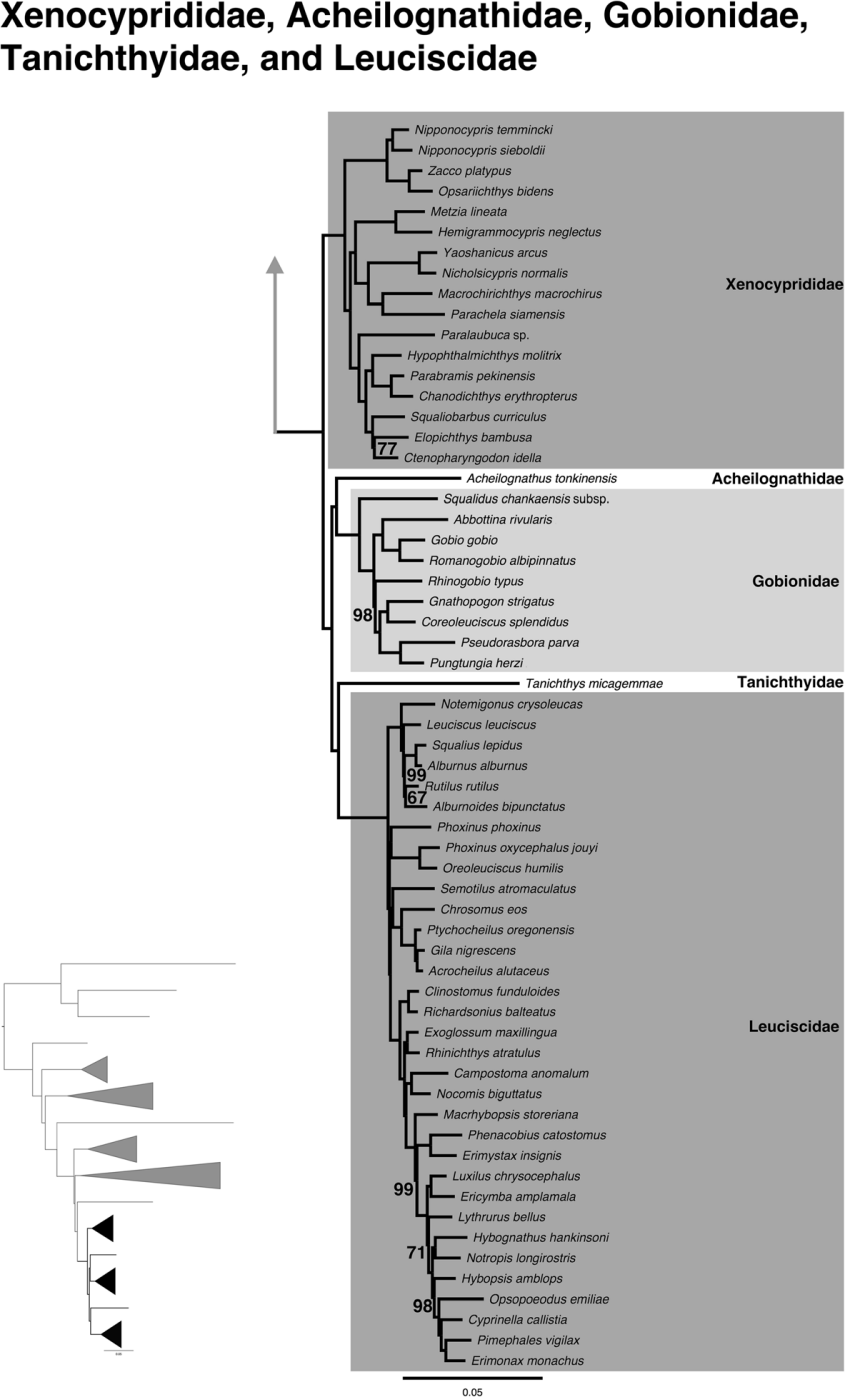
Expansion of Xenocyprididae, Acheilognathidae, Gobionidae, Tanichthyidae, and Leuciscidae from the ML tree shown in Fig. 1 (inset). All nodes are 100 % bootstrap supported unless otherwise indicated, and the scale bar represents the number of nucleotide substitutions per site

Placement of these families has varied across different studies [1, 4, 7, 69, 70] and here we obtain sister relationships between Acheilognathidae + Gobionidae and Tanichthyidae + Leuciscidae, with Xenocyprididae sister to all four of these families (Fig. 2). Within Xenocyprididae, relationships are similar to those found by Tao et al. [23] for the five taxa common to both studies (Fig. 6). This differs from relationships reported by He et al. [38] and Wang et al. [28], but the congruencies to Tao et al. [23] are not surprising given that their data were also acquired on a phylogenomic scale (100 genes, 13 taxa). Tang et al. [7] used two nuclear and two mitochondrial markers to elucidate the relationships among Xenocyprididae [9] (referred to as Oxygastrinae in their paper) and our results only differ for those relationships they obtained that were poorly supported. These include a different placement of the *Metzia* + *Hemmigrammocypris* clade and differing relationships among genera within a clade that includes *Hypophthalmichthys*, *Parabramis*, *Chanodichthys*, *Squaliobarbus*, *Ctenopharyngadon*, and *Elopichthys*. For Gobionidae, results in this study are highly congruent with previous molecular studies [4, 14, 71] that resolve the following clades and their relationships to each other: *Pseudogobio* group, *Gobio* group, *Sarcocheilichthys* group, and *Hemibarbus* group (see Yang et al. [71] for group designations). Leuciscidae has long been supported as monophyletic across many studies [1, 3, 19, 24–29, 31, 32, 72, 73] but relationships among the genera within have had differing results. Clades have been resolved in multiple studies and include: (1) far eastern phoxinins (Eurasian), (2) open posterior myodome (OPM), (3) creek chub – plagopterin (CC-P), (4) western North America (WNA), and (5) leuciscin (European) [4, 13, 24, 26, 28, 34, 74–81]. Our results also obtained the five major clades within Leuciscidae (Fig. 6), but yield strongly supported novel relationships that change our understanding of the biogeographical patterns exhibited by this family. Similar to the previous studies, we find *Notemigonus* (North American) within the leuciscin (European) clade, but in sharp contrast to these studies, all other North American Leuciscidae are monophyletic. This study provides a framework to further investigate the timing and number of invasions of leuciscids to North America. The hypothesized rapid diversification of North American leuciscids has led to difficulty in resolving relationships within this clade, but our robust phylogeny exemplifies the potential for anchored enrichment and next-generation sequencing in elucidating the relationships within problematic clades.

## Conclusions

The Cypriniformes is among the most important clades of freshwater fishes and among the most studied with phylogenetic inference. This great deal of work makes them a key group in understanding the various pit-falls of phylogenetic studies, and they exemplify the phylogenetic conflicts from the varying analyses of morphological, mitochondrial, and nuclear data. While many major clades of Cypriniformes have been long-supported, relationships within and among them have proven difficult to resolve across the entire order. Varying markers and morphological data have given different results and have been difficult to apply across such a large and diverse group. With the development of phylogenomic techniques, researchers can now acquire a substantial amount of highly informative, quality data for resolving dynamic relationships, and we demonstrate the efficacy of the approach using the very complex cypriniforms. Robust phylogenies are not only a prerequisite for a stable taxonomy, but are needed to address important evolutionary questions such as the timing of diversification, the geographic origins of clades, and the evolution of morphological and ecological novelty. For example, according to our results, Cypriniformes appear to have invaded North America at least twice and Africa several times from Eurasia, with these transcontinental migrations resulting in very diverse clades. With the robust phylogeny we present here, we provide a framework for studying the consequences of these transcontinental migrations and how clades can diversify from within established ecosystems. Such studies will have broad consequences in studies on the evolution of diversity.

The great diversity of Cypriniformes and the inclusion of perhaps the most important vertebrate model organism (Zebra Danio) make Cypriniformes an ideal group for comparative analyses. Considerable insight into the functioning of genes within vertebrate organisms has been obtained from the analysis of the Zebra Danio including forced mutations that often result in unviable larvae. By comparing the genome of the Zebra Danio with close relatives, the role of mutations and gene expression can be determined. Comparative genomic studies within Cypriniformes have already benefited from the foundation and annotation of the Zebra Danio genome sequence to generate insights into the functional evolution of various adaptations including adaptation to harsh environments such as caves and high altitude streams [82, 83]. With a robust phylogeny, we can get a much better understanding of the function of genes by treating relatives of the Zebra Danio as natural mutants screened by natural selection [1]. As the Cypriniformes continues to become a more genome-enabled clade, with several new genomes published in the last few years [83–86], we expect our phylogeny to provide a useful framework for comparative genomics.

## Additional files

Additional file 1:
**Table S1.** List of all tissue material used in this study. (PDF 66 kb)
Additional file 2:
**Table S2.** Summary of the sequence data collected and assemblies that resulted. (XLSX 91 kb)
Additional file 3:
**Figure S1.** Maximum likelihood tree for concatenated dataset of 172 ingroup and three outgroup taxa, fully expanded. (TIFF 11667 kb)
Additional file 4:
**Figure S2.** Species tree for all taxa, fully expanded, using STAR [51]. (TIFF 4588 kb)
Additional file 5:
**Figure S3.** Species tree for all taxa, fully expanded, using ASTRAL [53]. Internal branch lengths are in coalescent units and branches that lead to tips are not calculated by ASTRAL but instead arbitrarily displayed. (PDF 4611 kb)

## Abbreviations

Bp: Base pairs
CC-P: Creek chub – plagopterin
CT: Concatenated tree
OPM: Open posterior myodome
pp: Posterior probability
ST: Species tree
WNA: Western North America
