# Supplementary material for: Resolving Cypriniformes relationships using an anchored enrichment approach
Source: BMC Evol Biol. 2016 Nov 9;16:244. doi: 10.1186/s12862-016-0819-5 (PMC5103605; doi:10.1186/s12862-016-0819-5)
Supplement: Additional file 1: — Table S1. List of all tissue material used in this study. (PDF 66 kb) [file 12862_2016_819_MOESM1_ESM.pdf]

Table 1. Specimens used in this study. Authors of family group names are indicated with number of species in parentheses. Taxonomy assignments follow Mayden and Chen (2010), Tang et al. (2013), Kottelot (2013), van der Laan et al. (2014) and Yang et al. (2015) with one new subfamily, Eosominae, described herein. AUFT = Auburn University Fish Tissue Collection; UAIC =University of Alabama Ichthyological Collection; IHB = Institute of Hydrobiology, Chinese Academy of Sciences; SLUM = St. Louis University Museum; CTOL = Cypriniformes Tree of Life Project; NCMNS = North Carolina Museum of Natural Sciences.

Order	Suborder	**Family (# of spp.)**	Subfamily	Tribe	Species	Specimen Voucher
Outgroup						
Characiformes					*Pygocentrus nattereti*	AUFT 3043
Gymnotiformes					*Electrophorus electricus*	AUFT 3843
Siluriformes					*Callichthys callichthys*	AUFT 4774
Ingroup						
Cypriniformes						
	Cobitoidei					
		**Balitoridae Swainson 1839 (~92)**				
					Homaloptera ogilviei	SLUM B90.T114
		**Botiidae Borg 1940 (56)**				
					Sinibotia robusta	UAIC 14182.21
					Yasuhikotakia lecontei	AUFT 5061
		**Catostomidae Agassiz 1850 (83)**				
			Catostominae Agassiz 1850			
				Catostomini Agassiz 1850		
					Catostomus bernardini	SLUM 1558.04
					Catostomus cahita	SLUM 1562.03
					Catostomus leopoldi	SLUM MXSp09-674
					Catostomus platyrhynchus	AUFT 0183
					Catostomus plebeius	SLUM 1633.02
					Catostomus wigginsi	SLUM MXSp09-574
				Erimyzonini Hubbs 1930		
					Erimyzon oblongus	SLUM B21.T-1553
				Moxostomatini Bleeker 1863		
					Minytrema melanops	SLUM B46.T-4436
				Thoburnini Hubbs 1930		
					Thoburnia atripinnis	SLUM B21.T-1616
					Thoburnia rhothoeca	SLUM B21.T-1619
			Ictiobinae Bleeker 1863			
					Ictiobus niger	SLUM B78.10088
		**Cobitidae Swainson 1838 (198)**				
					Acantopsis sp.	UAIC 14310
					Cobitis biwae	CTOL 00224
					Lepidocephalichthys hasselti	CTOL 03230
					Misgurnus bipartitus	IHB 0411008
					Pangio anguillaris	SLUM B90.T139
		**Gastromyzontidae Fowler 1905 (137)**				
					Beaufortia kweichowensis	SLUM B90.T118
					Pseudogastromyzon myersi	UAIC 14169.22
		**Gyrinocheilidae Gill 1905 (3)**				
					Gyrinocheilus aymonieri	AUFT 5008
		**Nemacheilidae Regan 1911 (658)**				
					Lefua echigonia	SLUM B89.T006
					Nemacheilus corica	UAIC 14167.55
					Paracanthocobitis botia	CTOL 03287
					Schistura fasciolata	CTOL 00257
		**Vaillantellidae Nalbant and Bănărescu 1977 (26)**				
					Vaillantella maassi	CTOL 03437
	Cyprinoidei					
		**Acheilognathidae Bleeker 1863 (75)**				
					Acheilognathus tonkinensis	AUFT6614
		**Cyprinidae Rafinesque 1815 (1,623)**				
			Acrossocheilinae Yang et al. 2015			
					Acrossocheilus monticola	CTOL 00272
			Barbinae Bleeker 1859			
					Barbus barbus	UAIC 14167.25
					Capoeta aculeata	CTOL 03281
					Cyprinion semiplotum	CTOL 01499
			Cyprininae Rafinesque 1815			
					Carassioides acuminatus	SLUM B89.T037
					Cyprinus carpio	SLUM B89.T027
			Labeoninae Bleeker 1859			
					Akrokolioplax bicornis	SLUM B69.D8
					Barbichthys laevis	CTOL 02310
					Cirrhinus cirrhosus	SLUM B91.T178
					Cirrhinus microlepis	CTOL 01558
					Crossocheilus latius	CTOL 01569
					Crossocheilus reticulatus	CTOL 01561
					Garra flavatra	SLUM B91.T179
					Garra rufa	CTOL 03282
					Garra waterloti	CTOL 03174
					Gibelion catla	SLUM B90.T092
					Gymnostomus siamensis	CTOL 02856
					Labeo rohita	CTOL 01610
					Labeo senegalensis	CTOL 03175
					Labiobarbus leptochilus	CTOL 03347
					Lobocheilos melanotaenia	CTOL 01612
					Osteochilus vittatus	CTOL 01697
					Placocheilus dulongensis	SLUM B69.B6
					Schismatorhynchos nukta	CTOL 03180
			Poropuntiinae Menon 1999			
					Albulichthys albuloides	CTOL 01543
					Amblyrhynchichthys truncatus	CTOL 01545
					Barbonymus gonionotus	CTOL 01550
					Barbonymus schwanenefeldii	CTOL 01652
					Cosmochilus harmandi	CTOL 01560
					Cyclocheilichthys enolplos	CTOL 01495
					Discherodontus ashmeadi	CTOL 03207
					Mystacoleucus obtusirostris	CTOL 01618
					Poropuntius normani	CTOL 3918
					Sawbwa resplendens	SLUM B89.T050
			Probarbinae Yang et al. 2015			
					Catlocarpio siamensis	CTOL 01557
					Probarbus jullieni	CTOL 01623
			Schizopygopsinae Mirza 1991			
					Gymnodiptychus integrigymnatus	SLUM B69.DC0376
			Schizothoracinae McClelland 1842			
					Oreinus dulongensis	SLUM B69.B4
					Percocypris tchangi	SLUM B69.DC0344
			Smiliogastrinae Bleeker 1863			
					Chagunius chagunio	SLUM B90.T093
					Dawkinsia filamentosus	CTOL 01511
					Haludaria fasciata	UAIC 14169.14
					Hampala dispar	UAIC 14167.43
					Oreichthys cosuatis	UAIC 14167.48
					Pethia nigrofasciata	CTOL 01514
					Puntius sophore	SLUM B90.T121
					Rohtee ogilbii	CTOL 00449
			Spinibarbinae Yang et al. 2015			
					Spinibarbus caldwelli	CTOL 03193
			Torinae Karaman 1971			
					Labeobarbus compiniei	SLUM B90.T152
					Tor tambroides	UAIC 14182.02
		**Danionidae Bleeker 1863 (~330)**				
			Chedrinae Bleeker 1863			
					Chelaethips bibie	CTOL 03156
					Leptocypris niloticus	CTOL 03165
					Luciosoma setigerum	CTOL 01614
					Opsaridium ubangiense	AUFT 5799
					Opsarius koratensis	CTOL 03285
					Opsarius koratensis	AUFT 6617
					Opsarius pulchellus	SLUM B87.D5
					Opsarius tileo	AUFT 3793
					Raiamas senegalensis	AUFT 5433
					Salmostoma phulo	CTOL 00316
					Securicula gora	CTOL 03439
			Danioninae Bleeker 1863			
					Chela cachius	CTOL 00329
					Danio feegradei	CTOL 03198
					Danio margaritatus	AUFT 6618
					Danio rerio	Reference Genome
					Danio tinwini	AUFT 6619
					Danionella mirifica	CTOL 01954
					Danionella priapus	AUFT 6620
					Devario aequipinnatus	AUFT 6615
					Inlecypris auropurpurea	CTOL 01582
					Laubuka caeruleostigmata	CTOL 03205
					Laubuka laubuca	SLUM 06-060 (#081)
					Microdevario kubotai	UAIC 14166.24
					Microdevario nanus	CTOL 01616
					Microrasbora rubescens	CTOL 01583
					Neochela dadiburjori	CTOL 00330
			Esominae New Subfamily			
					Esomus danrica	AUFT 3811
			Rasborinae Günther 1868			
					Amblypharyngodon mola	SLUM B91.T198
					Horadandia atukorali	CTOL 01604
					Rasbora borapetensis	AUFT 6621
					Rasbora rubrodorsalis	UAIC 14175.07
					Trigonopoma pauciperforatum	AUFT 6622
		**Gobionidae Bleecker 1863 (206)**				
					Abbottina rivularis	CTOL 00259
					Coreoleuciscus splendidus	CTOL 01559
					Gnathopogon strigatus	CTOL 01759
					Gobio gobio	SLUM B12.T61
					Pseudorasbora parva	CTOL 00478
					Pungtungia herzi	CTOL 00483
					Rhinogobio typus	CTOL 00536
					Romanogobio albipinnatus	SLUM B12.T053
					Squalidus chankaensis	CTOL 01739
		**Leuciscidae Bonaparte 1835 (657)**				
					Acrocheilus alutaceus	AUFT 0194
					Alburnoides bipunctatus	CTOL 01752
					Alburnus alburnus	SLUM B12.T047
					Campostoma anomalum	AUFT 6108
					Chrosomus eos	AUFT 6624
					Clinostomus funduloides	AUFT 6616
					Cyprinella callistia	AUFT 6628
					Ericymba amplamala	AUFT 0033
					Erimonax monachus	NCMNS 61165
					Erimystax insignis	AUFT 6631
					Exoglossum maxillingua	AUFT 6627
					Gila nigrescens	SLUM B8.CBD09-04-01
					Hybognathus hankinsoni	AUFT 6625
					Hybopsis amblops	AUFT 6633
					Leuciscus leuciscus	SLUM B12.T33
					Luxilus chrysocephalus	AUFT 5982
					Lythrurus bellus	AUFT 0593
					Macrhybopsis storeriana	AUFT 0007
					Nocomis biguttatus	AUFT 6626
					Notemigonus crysoleucas	AUFT 6632
					Notropis longirostris	AUFT 0048
					Opsopoeodus emiliae	SLUM B43.T4247
					Oreoleuciscus humilis	CTOL 00446
					Phenacobius catostomus	AUFT 6629
					Phoxinus oxycephalus jouyi	CTOL 00469
					Phoxinus phoxinus	SLUM B91.T187
					Pimephales vigilax	AUFT 6630
					Ptychocheilus oregonensis	AUFT 0202
					Rhinichthys atratulus	SLUM B58.T6246
					Richardsonius balteatus	AUFT 0166
					Rutilus rutilus	SLUM B12.T041
					Semotilus atromaculatus	AUFT 5949
					Squalius lepidus	CTOL 03284
		**Paedocyprididae Mayden and Chen 2010 (3)**				
					Paedocypris cf. *progenetica*	AUFT 6623
		**Sundadanionidae Mayden and Chen 2010 (8)**				
					Sundadanio axelrodi “red”	CTOL 01723
		**Tanichthyidae Mayden and Chen 2009 (3)**				
					Tanichthys micagemmae	SLUM B91.T205
		**Xenocyprididae Günther 1868 (159)**				
					Aphyocypris normalis	CTOL 01619
					Chanodichthys erythropterus	SLUM 06-093
					Ctenopharyngodon idella	CTOL 00337
					Elopichthys bambusa	CTOL 03186
					Hemigrammocypris neglectus	CTOL 03199
					Hypophthalmichthys molitrix	CTOL 03276
					Macrochirichthys macrochirus	CTOL 01615
					Metzia lineata	SLUM B89.T58
					Nipponocypris sieboldii	CTOL 00604
					Nipponocypris temmincki	CTOL 00605
					Opsariichthys bidens	CTOL 00448
					Parabramis pekinensis	CTOL 00459
					Parachela siamensis	CTOL 03246
					Paralaubuca sp.	SLUM B87.TA5
					Squaliobarbus curriculus	CTOL 00569
					Yaoshanicus arcus	CTOL 01747
					Zacco platypus	CTOL 00602
