# Supplementary figures and images for: Resolving Cypriniformes relationships using an anchored enrichment approach
Source: BMC Evol Biol. 2016 Nov 9;16:244. doi: 10.1186/s12862-016-0819-5 (PMC5103605; doi:10.1186/s12862-016-0819-5)

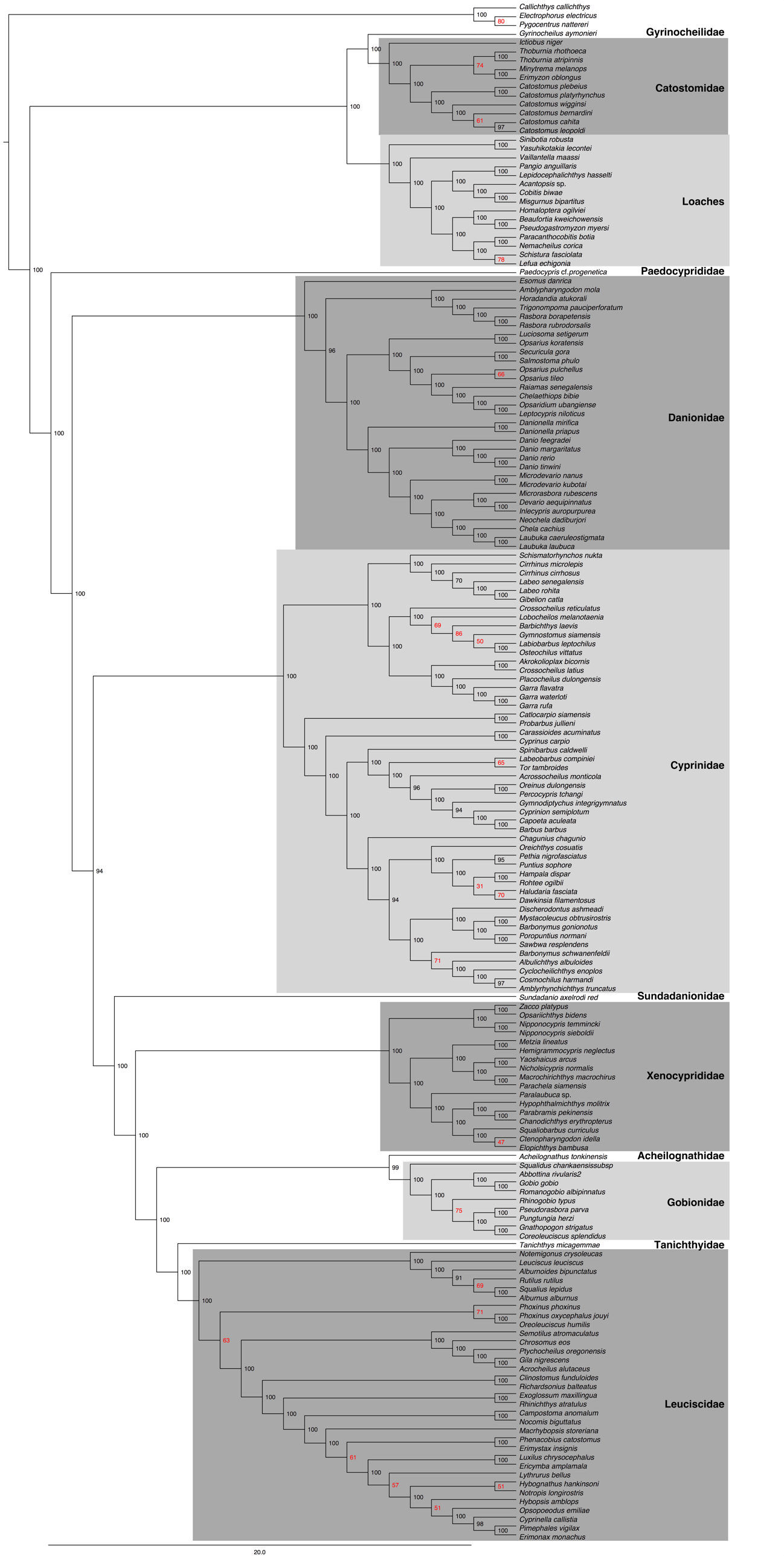

Supplement: Additional file 4: — Figure S2. Species tree for all taxa, fully expanded, using STAR [51]. (TIFF 4588 kb) [file 12862_2016_819_MOESM4_ESM.tiff]

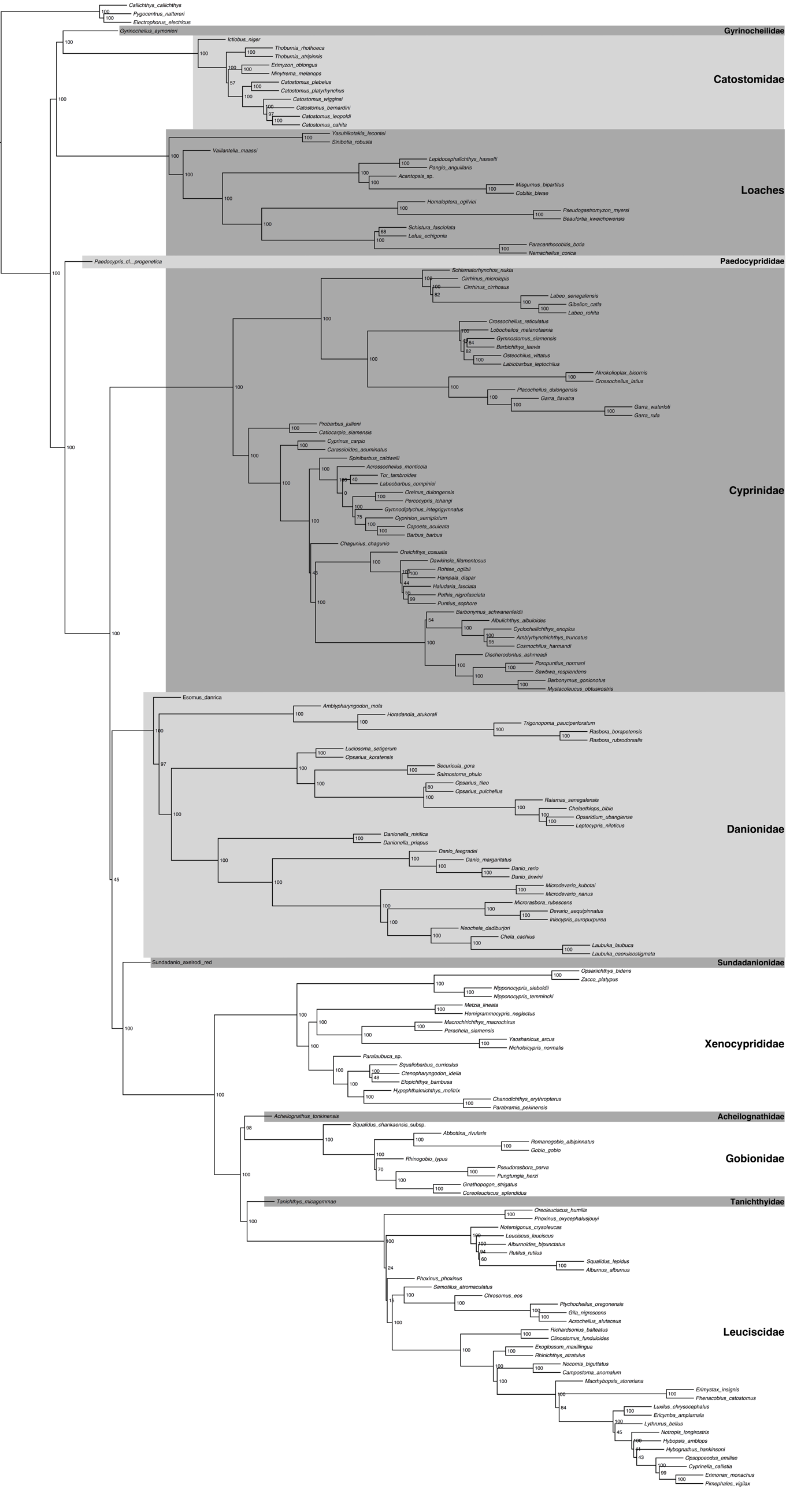

Supplement: Additional file 5: — Figure S3. Species tree for all taxa, fully expanded, using ASTRAL [53]. Internal branch lengths are in coalescent units and branches that lead to tips are not calculated by ASTRAL but instead arbitrarily displayed. (PDF 4611 kb) [file 12862_2016_819_MOESM5_ESM.pdf]
